# Immunomodulatory and antioxidant effects of total flavonoids of *Spatholobus suberectus* Dunn on PCV2 infected mice

**DOI:** 10.1038/s41598-017-09340-9

**Published:** 2017-08-17

**Authors:** Yuan-fang Fu, Li-he Jiang, Wei-dan Zhao, Meng Xi-nan, Shi-qi Huang, Jian Yang, Ting-jun Hu, Hai-lan Chen

**Affiliations:** 1 Animal Science and Technological College, Guangxi University, Nanning Guangxi, 530004 People’s Republic of China; 2 Department of Occupational Health, School of Medicine, Guangxi University, Nanning Guangxi, 530004 People’s Republic of China; 3 Guangxi Key laboratory of Metabolic Diseases Research, Guilin 181st Hospital, 541002 Guilin Guangxi, People’s Republic of China

## Abstract

Oxidative stress plays an important role in the pathogenesis of virus infection and antioxidants are becoming promising candidates as therapeutic agents. This study is designed to investigate the effect of total flavonoids of *Spatholobus suberectus* Dunn (TFSD) on oxidative stress in mice induced by porcine circovirus type 2 (PCV2) infection. The PCV2 infection leads to significant decrease in thymus and spleen indices, elevation of xanthine oxidase (XOD) and myeloperoxidase (MPO) activities, reduction in GSH level and GSH to GSSG ratio and decline of superoxide dismutase (SOD) activity, indicating the formation of immunosuppression and oxidative stress. TFSD treatment recovered the alteration of viscera index, antioxidant content and activities of oxidative-associated enzymes to a level similar to control. Our findings suggested that PCV2 induced immunosuppression and oxidative stress in mice and TFSD might be able to protect animals from virus infection via regulation of immune function and inhibition of oxidative stress.

## Introduction

Porcine circovirus type 2 (PCV2), one of the smallest DNA viruses that replicate autonomously in mammalian cells, is the main causative agent for PCV-associated diseases (PCVAD)^1, 2^. One or more clinical manifestations can be found from pigs with PCVAD, such as multisystemic wasting disease with weight loss, respiratory signs, enteric signs, high mortality and reproductive disorders^3^. Currently, PCV2 is considered as one of the most important pathogens for domestic swine, causing huge economic losses to the pig industry^4^. The PCV2 infection induce oxidative stress and immunosuppression^5^, represented by significant decrease in thymus and spleen indices, superoxide dismutase (SOD) activity, total antioxidant capacity (TAOC) and glutathione (GSH) level in the spleen and thymus^6^. Further, PCV2 induced oxidative stress and immunosuppresion can facilitate viruses replication and thus accentuate further diseases^7^.

Since late 2009, commercial vaccines against this virus have been used in pigs^8^. However, due to the complexity of PCV2 infection, the vaccine “might not work as expected” or “may fail”^8^. Trying to reduce the dependence on vaccines, there is a great demand for alternative methods to reduce the growth retardation and infection caused by PCV2. Administration of antioxidant molecules to regulate oxidative status in the organisms can be a potential novel pharmacological approach to control virus-induced inflammation and their long-term consequences^9^.

*Spatholobus suberectus* (*S. suberectus*) Dunn, a traditional Chinese medicinal herb, exhibits various pharmacological activities, including circulation invigoration^10^, anti-inflammatory^11^, anti-bacteria^12^ and antitumor activities^13^. There are many secondary compounds found in *S. suberectus* Dunn, but the major bioactive substances are flavonoids, including3′,4′,7-trihydroxyflavone, formononetin, calycosin, prunetin, eriodictyol, butin, liquiritigenin, plathymenin, dihydroquercetin, and dihydrokaempferol^14^. Flavonoids from *S. suberectus* Dunn showed potent inhibitory effect on 5-lipoxygenase (5-LOX), an enzyme critical for leukotriene biosynthesis^11^. In addition, significant inhibitory effect on HIV-1 protease activity has been reported from the aqueous extracts of *S. suberectus* ^15^. However, a limited number of studies have been conducted on the biological effects of the components of this herb on PCV-2 induced oxidative stress *in vitro* and *in vivo*. In our previous study, TFSD exhibited excellent regulative effect on PCV2 induced oxidative stress in RAW264.7 cells^16^. In the present study, the content of two major constituents in the total flavonoids of *S. suberectus* Dunn (TFSD) was firstly analyzed using high performance liquid chromatography (HPLC) and the regulatory effect of TFSD on PCV2 induced immunosuppression and oxidative stress in mice was further investigated.

## Results

### Determination of the content of formononetin and isoliquiritigenin in TFSD

**Figure 1:**
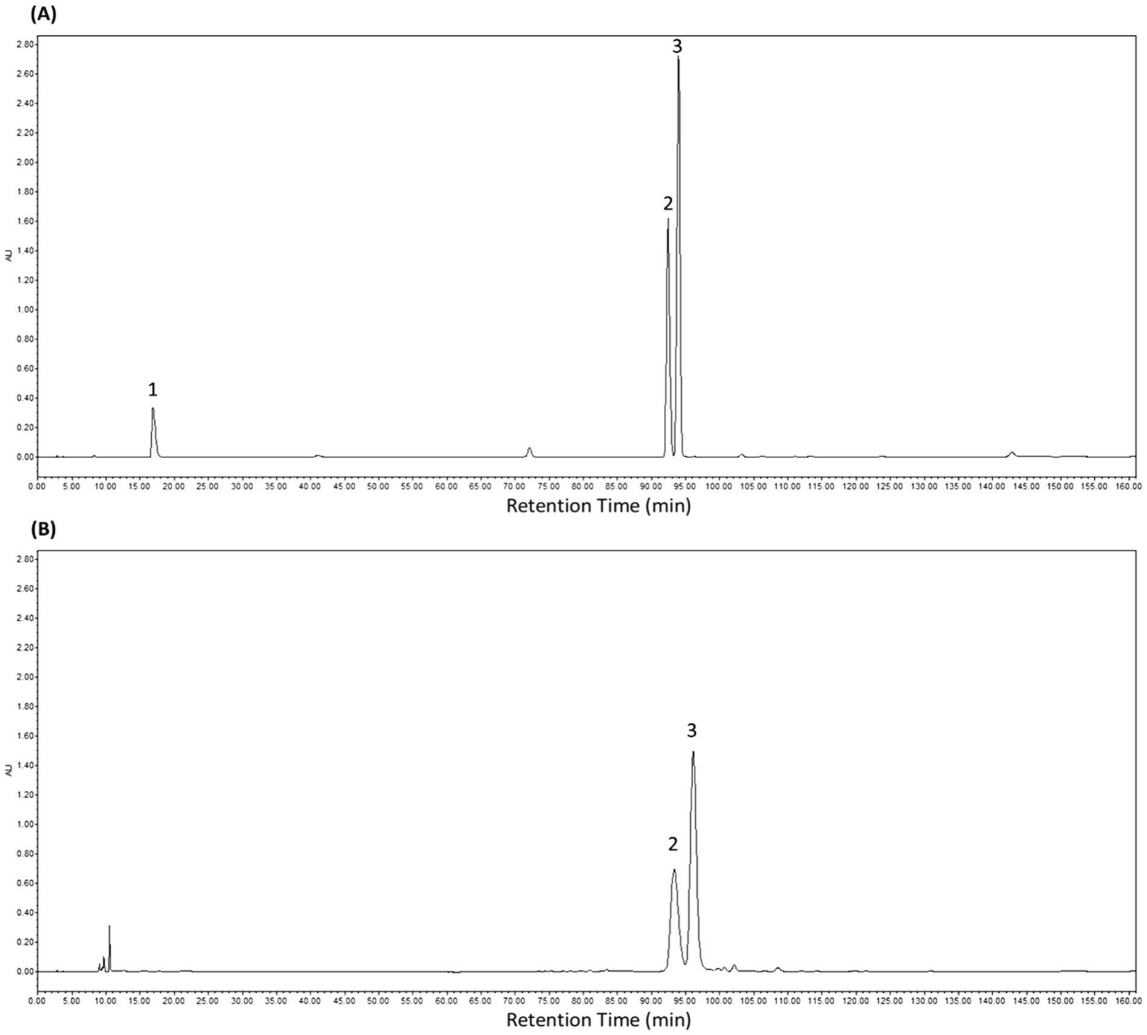
HPLC chromatograms of formononetin and isoliquiritigenin in TFSD. (**A**) Mixture of epicatechin, formononetin and isoliquiritigenin standards; (**B**) Analysis of formononetin and isoliquiritigenin in TFSD. Elution peaks: 1: epicatechin; 2: formononetin; 3: isoliquiritigenin.

**Figure 2:**
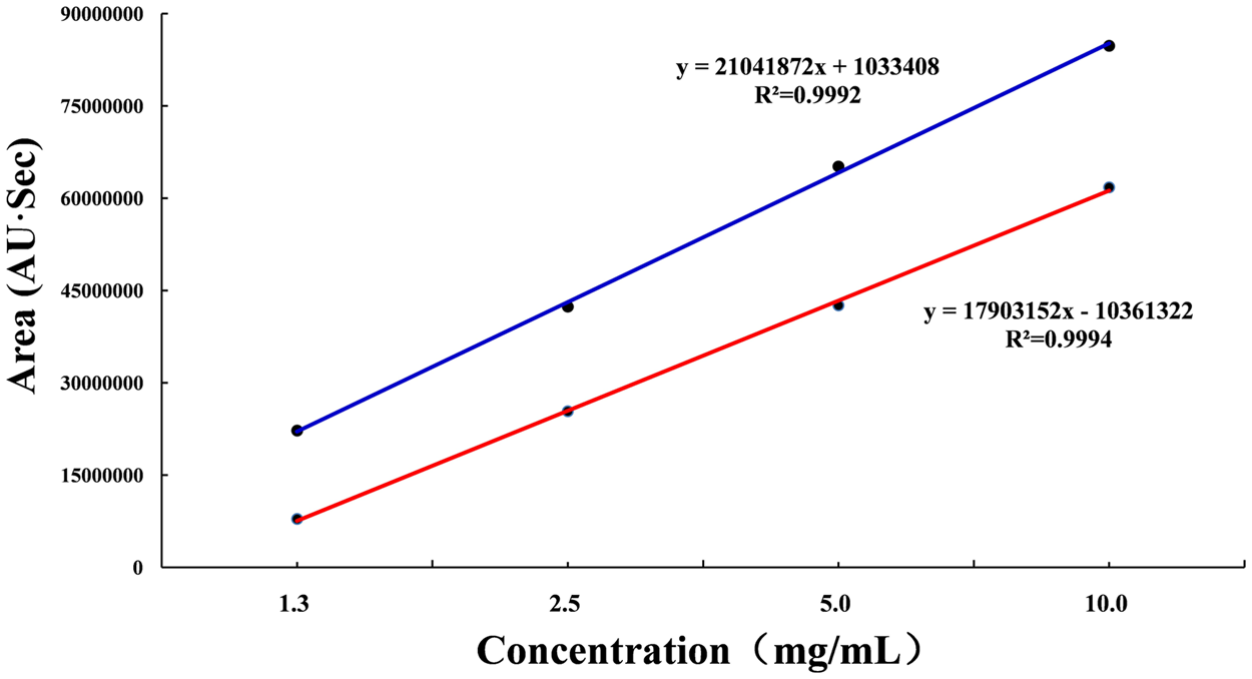
Standard curves of formononetin and isoliquiritigenin determined by HPLC.

From Fig. 1, it can be seen that isoflavones (formononetin and isoliquiritigenin) were clearly separated from other constituents in TFSD. By analyzing the peak areas under the curve at series concentrations, the standard curves for formonetin and isoliquiritienin were determined as y = 17903152x − 10361322 (R² = 0.9994) and y = 21041872x + 1033408 (R² = 0.9992), respectively (Fig. 2). The linear range for both formonetin and isoliquiritienin were 62.5 ~ 2000 μg/ml. According to the standard curves, the contents of formononetin and isoliquiritigenin in TFSD were determined to be 444.38 mg/g and 198.25 mg/g in TFSD, respectively. The analysis of formononetin and isoliquiritigenin content using HPLC method can be used as the quality control of TFSD in further studies.

### Detection of PCV2

**Figure 3:**
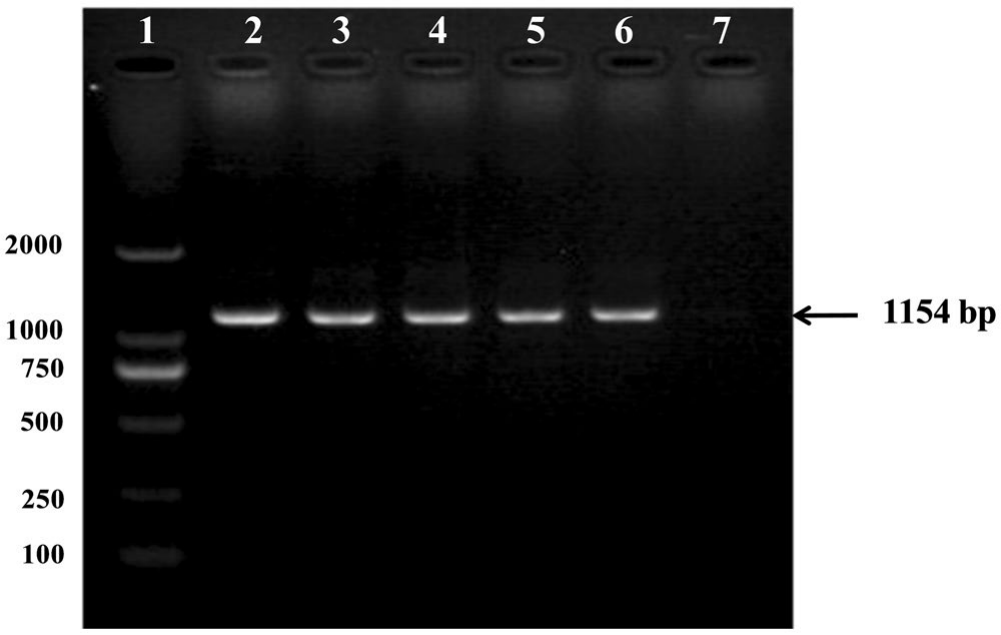
A specific nucleotide fragment of PCV2 was amplified via PCR method from the mouse spleen tissue. 1: DL2000 DNA marker; 2–5: spleen samples collected from PCV2 group; 6: positive control (virus dilution); 7: spleen sample from control mice without PCV2 infection.

A specific fragment of 1154 bp similar to positive control was detected from mice infected with PCV2, whereas samples collected from control group showed negative results (Fig. 3). These results indicated that PCV2 was able to invade and replicate in mice spleen.

### Effect of TFSD on the thymus and spleen indices

**Figure 4:**
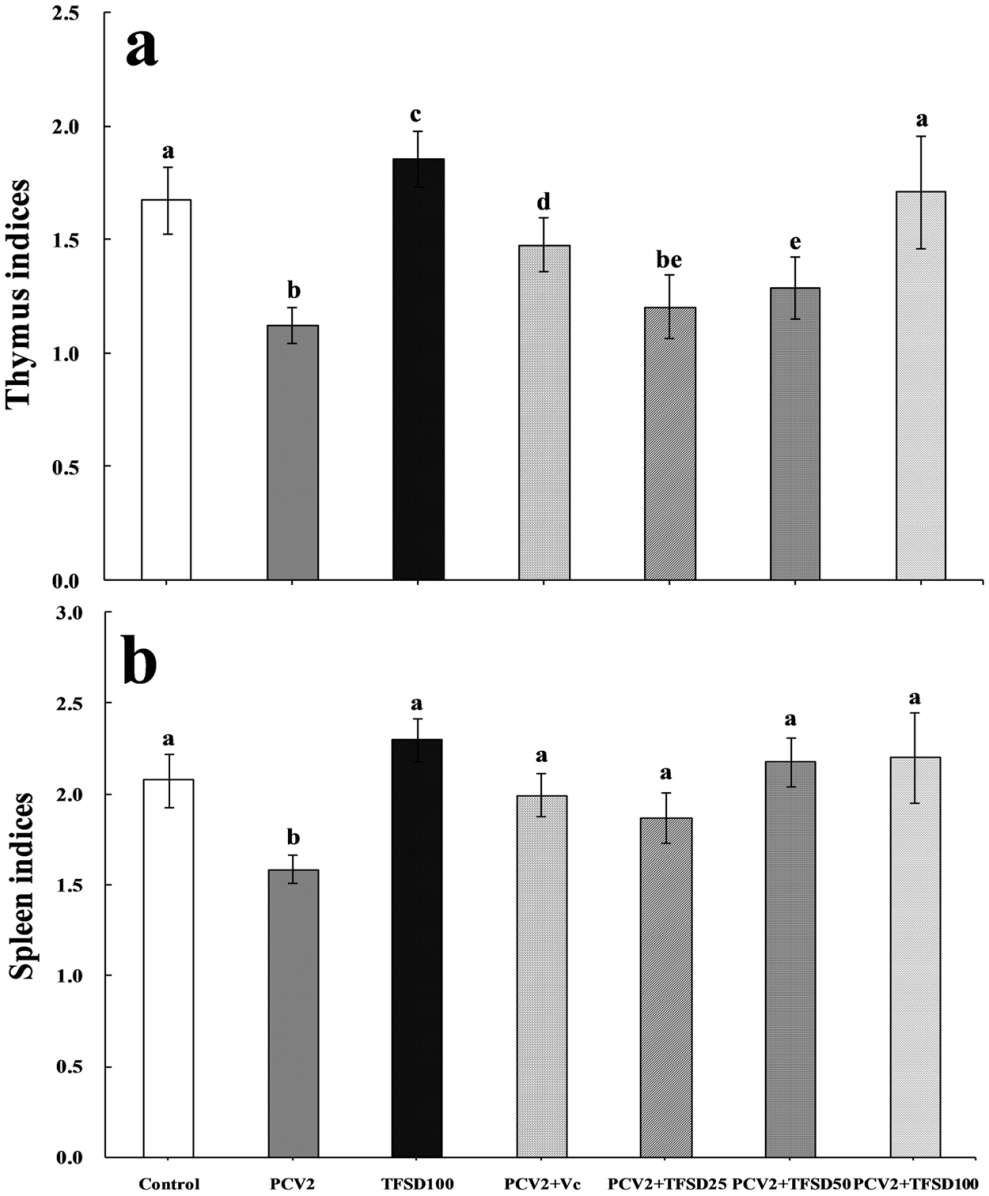
Effect of TFSD on the spleen and thymus indices of PCV2 infected mice. The spleen and thymus indices were calculated as the weight of spleen or thymus to the mice’s body weight (mg/g). Data are presented as mean ± SD (n = 10). Bars with different letters are statistically different (*P* < 0.05).

From Fig. 4, when mice were administrated with TFSD without PCV2 infection, the thymus index was remarkably increased (*P* < 0.05) and spleen index was also marginally improved (*P* < 0.05), indicating that TFSD itself will not damage but benefit the immune function of mice. However, the thymus and spleen indices were significantly decreased after PCV2 infection (*P* < 0.05), suggesting the compromise of immune function. Vitamin C (Vc) and TFSD treatment was able to compensate the reduction of thymus and spleen indices induced by PCV2 infection. Comparing to PCV2 group, 50 and 100 μg/mL TFSD treatment significantly increased the thymus index (*P* < 0.05), and 100 μg/mL TFSD was able to recover the thymus index to a level close to that of control (Fig. 4a). In addition, the spleen index in mice administrated with 25–100 μg/mL TFSD were dramatically increased compared to those without antioxidant treatment (*P* < 0.05). The obtained results clearly suggested that antioxidant administration after PCV2 infection was able to protect the immune functions from virus infection.

### Effect of TFSD on GSH and GSSG contents

**Figure 5:**
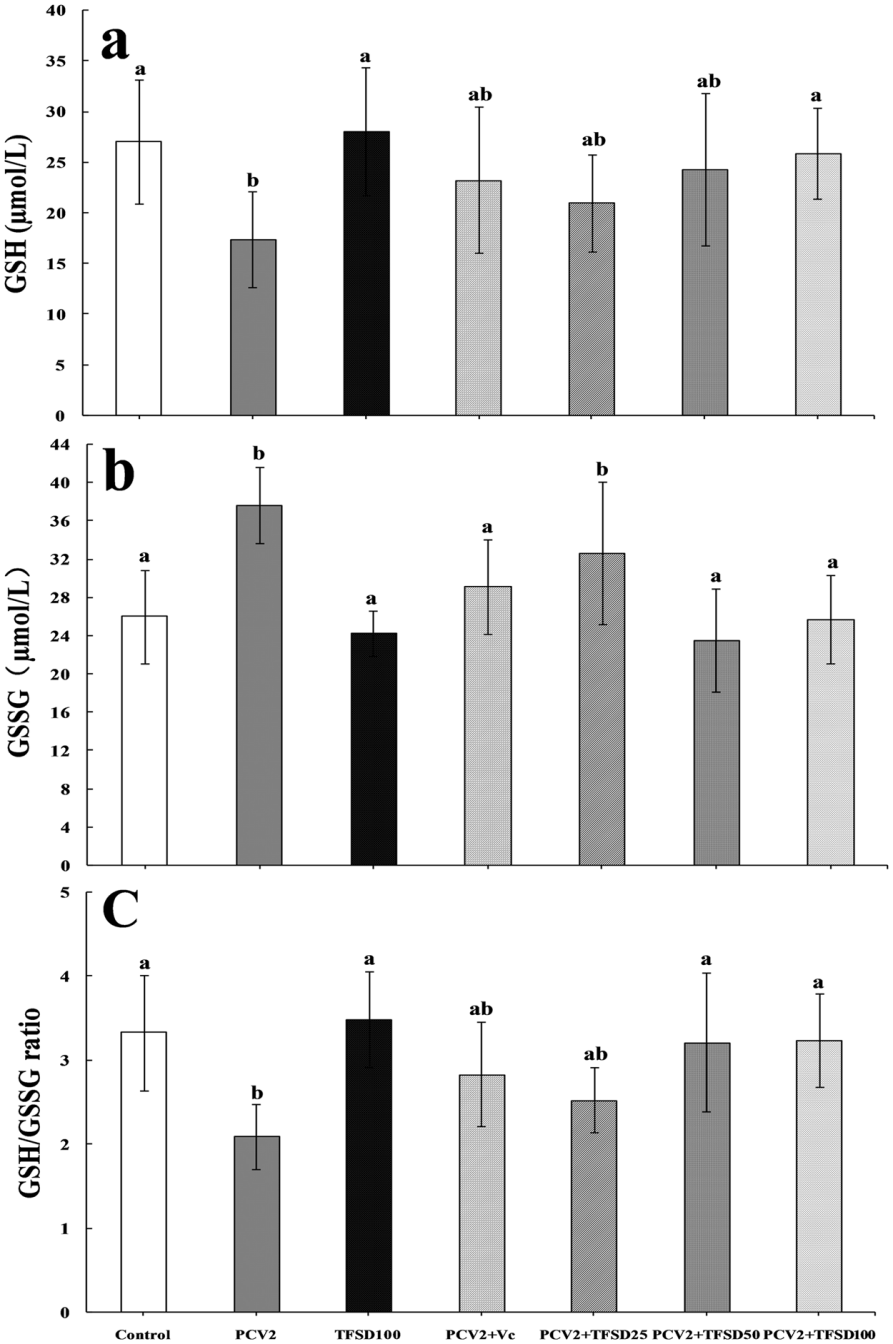
Effect of TFSD on GSH content, GSSG level and GSH to GSSG ratio in the spleen tissue of PCV2 infected mice. Data are presented as mean ± SD (n = 10). Bars with different letters are statistically different (*P* < 0.05).

From Fig. 5, mice treated with TFSD without PCV2 infection does not show any change in the oxidative stress indicators, such as GSH level, GSSG content and GSH to GSSG ratio. However, PCV2 infection induced significant decrease of GSH level and GSH to GSSG ratio (*P* < 0.05), as well as remarkable elevation of GSSG content (*P* < 0.05), indicating that oxidative stress was formed in the spleen tissue upon PCV2 infection. TFSD treatment post PCV2 infection was able to inhibit the changes of oxidative status, represented by significant increase in GSH content (Fig. 5a) and GSH to GSSG ratio (*P* < 0.05) (Fig. 5c), and dramatically decrease of GSSG level (*P* < 0.05) (Fig. 5b). Administration of 100 mg/kg.bw TFSD recovered GSH level, GSSG content and GSH to GSSG ratio to a level close to that of control, indicating that TFSD was able to regulate the oxidative stress caused by virus infection.

### Effect of TFSD on SOD activity

**Figure 6:**
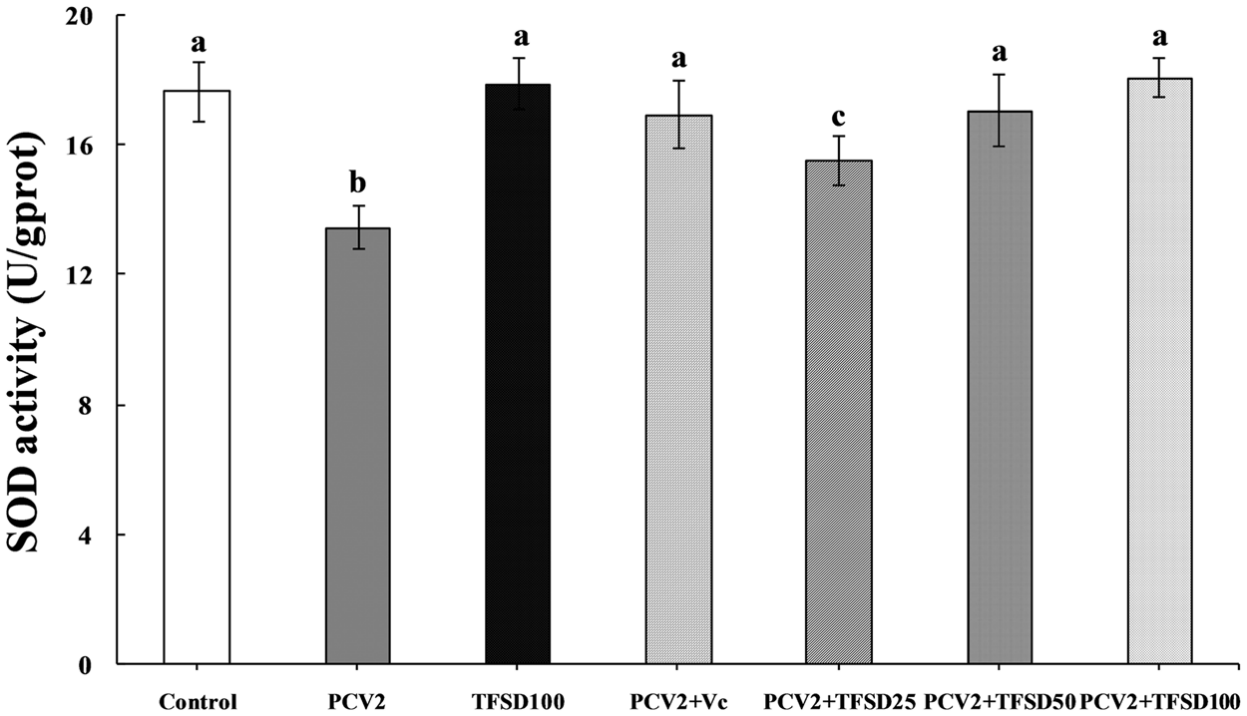
Effect of TFSD on SOD activities in the spleen of PCV2 infected mice. Data are presented as mean ± SD (n = 10). Bars with different letters are statistically different (*P* < 0.05).

From Fig. 6, SOD activity in the mice spleen tissue was not affected by TFSD itself, but was significantly inhibited by PCV2 infection (*P* < 0.05). Treatment with TFSD at 50 and 100 mg/kg.bw post virus infection remarkably increased the SOD activity in the spleen tissue in PCV2 infected mice (*P* < 0.05).

### Effects of TFSD on XOD and MPO activities

**Figure 7:**
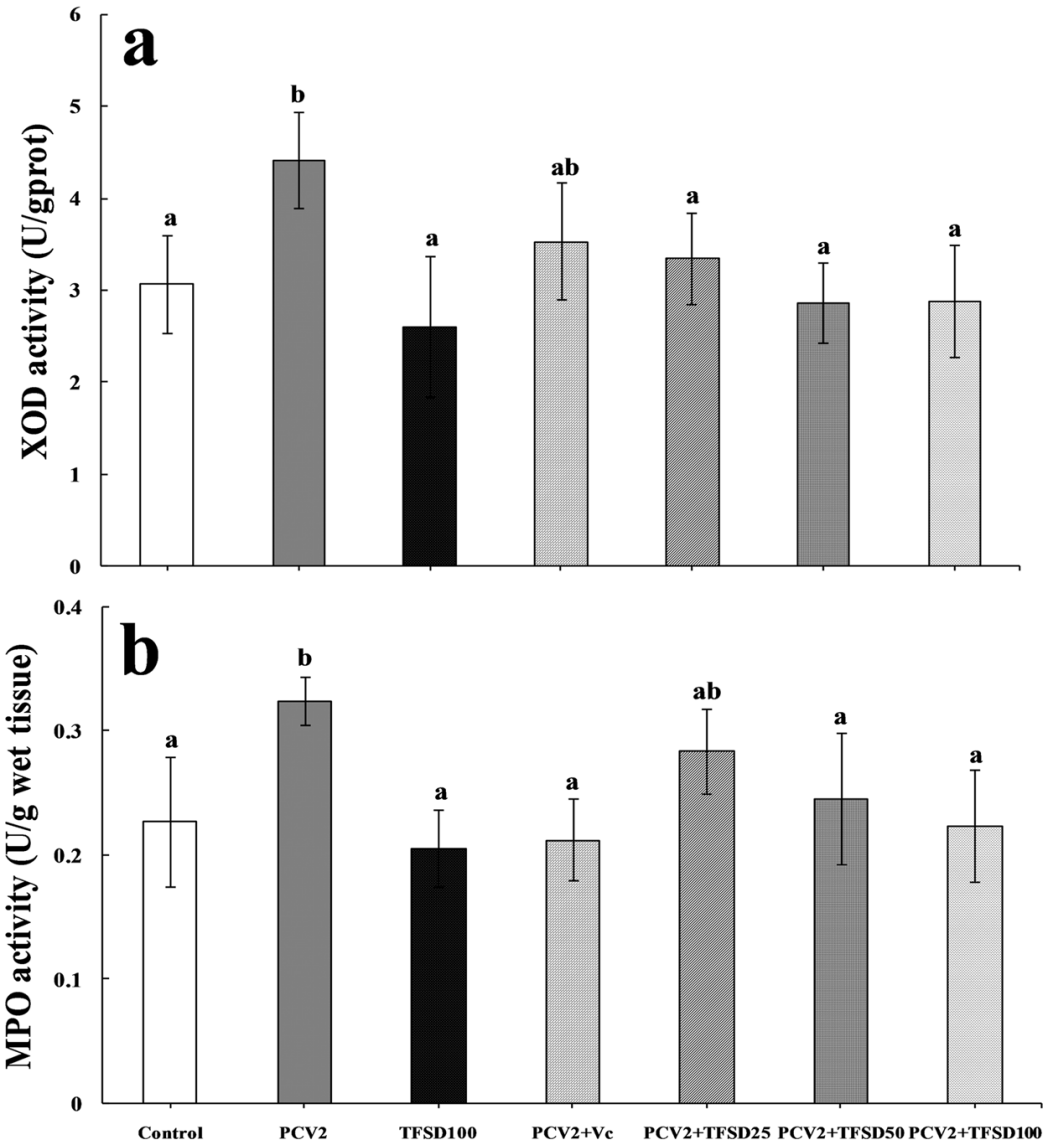
Effect of TFSD on XOD and MPO activities in the spleen of PCV2 infected mice. Data are presented as mean ± SD (n = 10). Bars with different letters are statistically different (*P* < 0.05).

TFSD treatment in mice without PCV2 infection did not caused any change of XOD and MPO activities in the spleen tissue while PCV2 infection resulted in significant increase of enzyme activities (*P* < 0.05) (Fig. 7), suggesting that oxidative status alteration was induced by PCV2 rather than TFSD. Administration of TFSD post PCV2 infection was able to promote the activity of XOD and MPO. Doses of 50 and 100 mg/kg.bw TFSD were able to recover the XOD and MPO activities to a level similar to control, suggesting that antioxidant can protect the immune functions from oxidative stress induced by PCV2 infection.

## Discussion

PCV2 is prominently consider as a main pathogen of PCVAD and previous studies reported that PCV2 could infected mice and replicated in the organism via intraperitoneal, intranasal or oral administrations^6, 17, 18^. In this study, a PCV2-specific 1154 bp nucleotide fragment similar to positive control, was amplified in the spleen tissue of mice infected with PCV2 (Fig. 3), indicating that PCV2 infected mouse model was successfully formed. PCV2 infection might lead to immunosuppression by depleting lymphocytes in the lymph nodes or spleen, causing superinfection and complicated syndromes^19^. The obtained results indicated that immunosuppression was formed in PCV2 infected mice, represented by significant decrease of both spleen and thymus indices compared to the control (*P* < 0.05). The TFSD treatment was able to increase the spleen and thymus indices and promote the function of immune organs, suggesting that TFSD can overcome the virus induced immunosuppression.

Reactive oxygen species (ROS) is considered as a double-edged sword, since low ROS level is required for cellular signaling cascades including those participated in the regulation of the antiviral and proinflammatory responses^20^, while excessive ROS production during pathogen infection often results in tissue damage and pathogenesis^21^. Viral infections often induce increased generation of ROS, causing alternation of redox homeostasis^22–24^. The failure to maintain an appropriate redox balance usually resulted in oxidative stress, which contributes to viral pathogenesis through alterations of biological structures, impaired of immune function, increased of viral replication and activation of inflammatory response^25, 26^. Fox example, oxidative stress can be stimulated by hepatitis C virus (HCV)^24^ and dengue virus^27^, leading to production of inflammatory cytokines and increase of disease severity. Time-dependent increase of ROS following PCV2 infection has been reported^28^ and oxidative stress induced by H_2_O_2_ enhanced PCV2 replication in PK-15 cells^28^. Decreased levels of SOD, catalase, glutathione peroxidase (GPx) and glutathione S-transferase (GST) were observed at a later time of PCV2 infection^29^. GSH depletion with buthioninesulfoximine (BSO) resulted in elevation of ROS levels and increase of PCV2 replication^28^. It was suggested that PCV2 infection might be promoted by ROS-induced NF-κB activation, as inhibiting NF-κB activity can inhibit the BSO-mediated increase of PCV2 replication^28^. Further, PCV2 infection induced release of proinflammatory factors such as IL-1β, IL-10, IL-8 and TNF-α, leading to decrease of cell viability^30^. In this study, increase of XOD and MPO activities, decrease of GSH level and GSH to GSSG ratio and decline of SOD activity in spleen tissue were observed upon PCV2 infection, indicating the formation of oxidative stress in mice.

Antioxidant mechanisms play a critical role on controlling antiviral and cell death responses to the virus. Increasing evidence showed that antioxidants act as protectors of host organism against infections^31^. For instance, respiratory syncytial virus induced clinical disease and pulmonary inflammation in mouse models were ameliorated by antioxidant treatment^32^. Exogenous GSH was reported to inhibit viral entry and prevented ROS accumulation in dengue virus infected HepG2 cells^9^. N-acetyl-l-cysteine (NAC) treatment was able to inhibit PCV2 replication inside the kidney cells^28^. Carboxymethylpachymaran (CMP) was able to protect mice from PCV2 induced damage by improving their immunity and antioxidant capacity^6^. Further, over expression of pig selenoprotein S, a protein possessing strong antioxidant ability, was able to block ochratoxin A induced promotion of PCV2 replication by inhibiting oxidative stress^33^. Astragalus polysaccharides, a traditional Chinese medicine showing antioxidant and immuno-modulatory activities, attenuated PCV2 infection by inhibiting oxidative stress and endoplasmic reticulum stress and by blocking NF-κB pathway^34, 35^. TFSD, possessing excellent anti-inflammatory, antioxidant activity and free radical scavenging ability, was speculated to be great candidate for the prevention of PCV2 induced oxidative damages.

The ratio of intracellular GSH to GSSG was consider as an important indicator for cell antioxidant capacity, and antioxidant enzymes were the primary defense that prevents biological macromolecules from oxidative stress induced damage^36^. SOD catalyzes the dismutation of superoxide radicals to protect biomacromolecules against oxidative processes initiated by the superoxide anion. MPO catalyze H_2_O_2_-mediated oxidation of halide ions and produce hypohalous acids (HOCl). Excess HOCl can oxidize biomacromolecules and induce damage to normal tissue. XOD is the enzyme responsible for the metabolism of hypoxanthine and xanthine to produce uric acid and superoxide radicals. The superoxide radicals can attack biomacromolecules, causing cell damage and producing excess ROS. In the current study, TFSD treatment was able to increase GSH level and the ratio of GSH to GSSG, raise the SOD activity and reduce MPO and XOD levels, suggesting that TFSD acquired an antioxidant effect, which can recover the intracellular oxidative status to protect mice from damages caused by PCV2 infection.

In summary, PCV2 induced significant immunosuppression and oxidative stress in the spleen of infected mice, as indicated by the decrease of both spleen and thymus indices, suppression of GSH, reduction of GSH to GSSG ratio, decrease of SOD activity and increase of XOD and MPO levels. Interestingly, the treatment of infected mice with TFSD dramatically increased the spleen and thymus indices, GSH level, GSH to GSSG ratio and SOD activity, and inhibited the MPO and XOD activities. Our results strongly suggests that TFSD plays a key role in overcoming the immunosuppression and oxidative stress caused by PCV2 infection, and can be an antioxidant candidate for the prevention and treatment of oxidative stress associated diseases, including disease caused by virus infection.

## Methods

### Reagent

Vitamin C, dimethylsulfoxide (DMSO), sulphanilamide, phosphoric acid (H_3_PO_4_), ethylene diamine tetraacetic Acid (EDTA), o-Phthalaldehyde (OPA), and n-ethylmaleimide (NEM) were purchased from Sigma, USA. Commercial kits for the analysis of superoxide dismutase (SOD), xanthine oxydase (XOD) and myeloperoxidase (MPO) were purchased from Nanjing Jiancheng Bioengineering Institute, China. QlAamp DNA Mini Kit was obtained from Qiagen, China. 2×Taq PCR MasterMix for PCR amplication was purchased from CWBIO, China. Primers for PCR amplification were synthesized by Sangon Biotech, Shanghai, China.

### TFSD preparation and HPLC analysis

*S. suberectus* Dunn which was collected in Chongzuo, Guangxi province, China in 2014, was purchased from the Chinese herbal medicine market in Zhongyao road in Nanning, Guangxi province. It was identified by Professor Hu Tingjun in the lab of pharmacology at Animal Science and Technology College, Guangxi University. The 30-fold volume of 50% ethanol was used to extract the total flavonoids of *S. suberectus* Dunn (TFSD) under 80 °C for 3 h. The ethanol extract was filtered in hot and evaporated to remove all ethanol using the rotary (evaporator Rotavapor R-3, BUCHI Labortechnik AG, Switzerland). The TFSD was purified by re-precipitating in 70% ethanol overnight for three times. The final extraction was dried using a vacuum freeze drier (Alpha 1-4LD Plus, Christ, Germany). 10 mg TFSD was dissolved in 1 mL of 0.1 M PBS (pH 7.2) containing 1% of DMSO and filtered with a 0.22 μm membrane to prepare the stock solution with a concentration of 10 mg/mL for further used.

A Waters 2695 separation module (Waters, Milford, MA) fitted with a Waters 2998 photodiode array detector (PDA, wavelength range: 210–400 nm) and an auto sampler was used to quantify the composition of TFSD and determine the content of formononetin and isoliquiritigenin in TFSD. Separation of formononetin and isoliquiritigenin was carried out using a Phenomenex Luna C18 reversed-phase column (Phenomenex, Aschaffenburg, Germany). The eluting compounds were detected at a wavelength of 260 nm. The mobile phase involved 100% methyl cyanides (A) and 0.1% acetic acid in water (v/v) (B). The elution conditions were 0–50 min 10–15% A, 50–70 min 15–25% A, 70–105 min 25–45% A, 105–125 min 45–65% A, 125–145 min 65–80% A. Additional chromatographic parameters such as flow rate was 1.0 mL/min and injection volume was 10 μL. Quantitative standards of formononetin and isoliquiritigenin were used to construct calibration plots for the determination of their content in TFSD samples. Each standard was prepared at four different concentrations in methanol, and analyzed using established method. The composition of TFSD and the content of formononetin and isoliquiritigenin in TFSD was determined by the retention time and the absorbance spectra using the calibration plots.

### Virus

Porcine circovirus type 2 (PCV2) was provided by the Laboratory of Preventive Veterinary at Animal Science and Technological College of Guangxi University and amplified using PK-15 cells. The viral titers were determined to be 10^4.7^ TCID_50_/0.1 mL using the Reed-Muench assay and diluted with the culture medium to 10^3.0^ TCID_50_ for the experiments.

### Infection of mice with PCV2 and experimental design

Kunming inbred mice of either sex, weighing 22 ± 2 g, were obtained from the Animal Center of Guangxi Medical University. Mice were acclimatized at an environment with a temperature of 24–26 °C and humidity of 60% for 3 days before experiment. Animals are handled in accordance with the animal ethics guidelines approved by the Animal Experimentation Ethics Committee (AEEC) at Guangxi University of China.

80 mice were randomly divided into eight groups with 10 mice per group according to Table 1. Mice were inoculated with 1 mL of PCV2 virus dilution via both intragastric administration (0.5 mL) and intraperitoneal injection (0.5 mL) daily for three days. PBS instead of virus dilution was used in control group and TFSD group. From Day 4 to Day 6, mice were intragastric administrated with either PBS (control group and PCV2 group) or antioxidants (Vc or TFSD) according to Table 1. On Day 7 post-inoculation, mice were weighed and sacrificed. The spleen and thymus were collected and immediately weighed for the calculation of spleen and thymus indices according to the formula that spleen or thymus index (mg/g) = (weight of spleen or thymus/body weight).

**Table 1:** Grouping and treatment of mice.

Group	Day 1–3	Day 4–6
Control	PBS 1 mL/mouse	PBS 0.02 mL/g.bw
PCV2	PCV2 1 mL/mouse	PBS 0.02 mL/g.bw
TFSD100	PBS 1 mL/mouse	TFSD 100 mg/kg.bw
PCV2 + Vc	PCV2 1 mL/mouse	Vc 100 mg/kg.bw
PCV2 + TFSD25	PCV2 1 mL/mouse	TFSD 25 mg/kg.bw
PCV2 + TFSD50	PCV2 1 mL/mouse	TFSD 50 mg/kg.bw
PCV2 + TFSD100	PCV2 1 mL/mouse	TFSD 100 mg/kg.bw

^*^Mice were inoculated with PCV2 via intragastric administration (0.5 mL/mouse) and intraperitoneal injection (0.5 mL/mouse) daily for three days. Mice were treated with PBS, Vc or TFSD via intragastric administration on Day 4, 5 and 6.

### Polymerase chain reaction (PCR) detection of PCV2 in spleen tissue

Spleen tissue samples were randomly obtained from 4 infected mice. The forward and backward primers for PCV2 quantitation were 5′-CCGCGGGCTGGCTGAACTT-3′ and 5′-ACCCCCGCCACCGCTACC-3′, respectively. Total DNA was isolated from spleen tissue using QlAamp DNA Mini Kit (QIAGEN, China) following the manufacture’s instruction. PCV2 gene specific fragment was amplified using PCR and segment of 1154 bp was detected via electrophoresis using 1% agarose gels containing 0.5 μg/mL ethidium bromide.

### Analyzing of GSH and oxidized glutathione (GSSG) content in spleen tissue

Homogenates of mice spleen were prepared using cold physiological saline (1:9, w/v ratio) and centrifuged at 5000 rpm, 4 °C for 10 min. The supernatant was collected for the analyzing of GSH and GSSG content. Briefly, 3.6 mL phosphate-EDTA buffer (pH 8.0) and 200 μL OPA (1 mg/mL) were added into 200 μL of supernatant and incubated for 40 min at room temperature. Fluorescent intensity was measured using an automatic microplate reader at an excitation wavelength of 350 nm and the corresponding emission wavelength of 425 nm. For the GSSG analysis, 40 μL NEM (0.04 mol/L) was added into another 100 μL of supernatant and incubated for 30 min at room temperature. 1.9 ml NaOH (0.1 mol/L) and 100 μL OPA (1 mg/mL) were added into the mixture and incubated for another 15 min at room temperature. Fluorescent intensity was measured with excitation wavelength of 337.8 nm and the emission wavelength of 421.6 nm. GSH and GSSG concentrations were determined by GSH and GSSG standard curves.

### Determination of SOD, XOD and MPO activities in spleen tissue

The supernatant prepared in section 2.6 was also used to determine the activities of intracellular superoxide dismutase (SOD), xanthine oxidase (XOD) and myeloperoxidase (MPO) using commercial kits according to the manufacturer’s instructions.

### Statistical analysis

Statistical analysis was performed using the software of SPSS version 17.0. Data were analyzed using one-way analysis of variance (ANOVA) followed by the Duncan test. Data were expressed as means ± S.D. Differences were regarded as significant at *P* < 0.05.

### Data availability

The datasets generated and analyzed during the current study are available from the corresponding author on reasonable request.
